# Development and Evaluation of Solid Lipid Nanoparticles of Raloxifene Hydrochloride for Enhanced Bioavailability

**DOI:** 10.1155/2013/584549

**Published:** 2013-10-20

**Authors:** Anand Kumar Kushwaha, Parameswara Rao Vuddanda, Priyanka Karunanidhi, Sanjay Kumar Singh, Sanjay Singh

**Affiliations:** Department of Pharmaceutics, Indian Institute of Technology (Banaras Hindu University), Varanasi 221005, India

## Abstract

Raloxifene hydrochloride (RL-HCL) is an orally selective estrogen receptor modulator (SERM) with poor bioavailability of nearly 2% due to its poor aqueous solubility and extensive first pass metabolism. In order to improve the oral bioavailability of raloxifene, raloxifene loaded solid lipid nanoparticles (SLN) have been developed using Compritol 888 ATO as lipid carrier and Pluronic F68 as surfactant. Raloxifene loaded SLN were prepared by solvent emulsification/evaporation method, and different concentrations of surfactant, and homogenization speed were taken as process variables for optimization. SLN were characterized for particle size, zeta potential, entrapment efficiency, surface morphology, and crystallinity of lipid and drug. *In vitro* drug release studies were performed in phosphate buffer of pH 6.8 using dialysis bag diffusion technique. Particle sizes of all the formulations were in the range of 250 to 1406 nm, and the entrapment efficiency ranges from 55 to 66%. FTIR and DSC studies indicated no interaction between drug and lipid, and the XRD spectrum showed that RL-HCL is in amorphous form in the formulation. *In vitro* release profiles were biphasic in nature and followed Higuchi model of release kinetics. Pharmacokinetics of raloxifene loaded solid lipid nanoparticles after oral administration to Wistar rats was studied. Bioavailability of RL-HCL loaded SLN was nearly five times than that of pure RL-HCL.

## 1. Introduction

Raloxifene hydrochloride (RL-HCL) is a nonsteroidal drug which comes under the classification of selective estrogen receptor modulator (SERM) having estrogenic effects on bone and antiestrogenic actions on endometrium and breast [1]. Oral dose of raloxifene (60 mg) has been approved for the prevention and treatment of postmenopausal osteoporosis once a day regimen. Raloxifene exerts its action by altering gene transcription and binding to intranuclear estrogen receptor [2]. It has been shown to reduce the risk of vertebral fractures from 4.3% to 1.9% and increases bone mineral density of the spine, hips, and total body by 2.1–2.6% after 36 months of treatment [3]. Even though, 60% of raloxifene is absorbed orally, the absolute plasma bioavailability is only 2% because of its poor aqueous solubility (0.25 mg/lit in water) and extensive first pass metabolism by glucuronide conjugation [4]. RL-HCL comes under class II (low solubility and high permeability) of biopharmaceutical classification system (BCS) with lesser bioavailability [5].

Hepatic first pass metabolism can be avoided by administering the drug through transdermal, buccal, rectal, and parenteral routes. Lymphatic delivery is an alternative approach to avoid the first pass metabolism of drugs in peroral drug delivery, and thereby improves bioavailability by diverse physiological actions which includes delaying gastric emptying time [6] and membrane lipid fluidity enhancement [7] or by directly draining into thoracic duct, further in to the venous blood [8] and inhibiting efflux transporters like p-glycoprotein (P-gp) [7]. The main function of the lymphatic system is to facilitate absorption of long chain fatty acids via chylomicron formation [9]. Targeting to intestinal lymphatic system through per oral delivery can be attained by any one of the following ways: (a) the use of absorption enhancers which opens the paracellular route, resulting in increased absorption of hydrophilic macromolecules or macromolecular conjugates [10], (b) via gut associated lymphoid tissue (GALT), and (c) via the intestinal lipid transport system; this is the major mechanism of lymphatic delivery of lipophilic compounds when formulated with lipid-based carriers such as microemulsions, liposomes, solid lipid nanoparticles (SLN), and modified lipoprotein [11, 12]. In recent years, great attention has been focused on lipid-based formulations to improve the oral bioavailability of poorly water soluble drugs [13].

SLN are the advanced drug delivery system in which the active ingredient is incorporated into lipid carrier (e.g., triglycerides, partial glycerides, fatty acids, steroids, and waxes) and stabilized by using biocompatible surfactants (e.g., poloxamer, polysorbate, sodium glycocholate, soybean lecithin) [14]. SLN have particle size in the submicron range (50 to 1000 nm) and are in solid state at physiological and room temperature [15]. Due to their smaller particle size, SLN offers larger surface area, prolonged release of drug and rapid uptake by cells [16]. Moreover, it has the feasibility to encapsulate drugs with different physiochemical and pharmacological properties. SLN have potential to overcome the solubility and bioavailability problem of the drug that has poor solubility and/or permeability [17].

SLN are composed of different type of lipid core that may mimic chylomicrons formation, which ultimately takes the carrier along with the entrapped drug by following the classical transcellular mechanism of lipid absorption [12]. This delivery system is suitable for lipophilic drug where aqueous solubility of the drug is the limiting factor for its absorption [14]. SLN promotes the oral absorption of poorly water-soluble lipophilic drugs and enhances the bioavailability [18].

Even though several research works are being carried out to enhance the solubility and bioavailability of raloxifene by formulating it as tablets [19], microemulsions [20], microspheres [5], transdermal patch [21], and polymeric nanoparticles [22], still it is required to design the formulation having combined advantages like sustained release and avoiding first pass metabolism of RL-HCL. Therefore, the aim of the present work is to improve the bioavailability of RL-HCL by formulating it as SLN. RL-HCL loaded SLN were prepared by solvent emulsification/evaporation method using Compritol 888 ATO (glyceryl behenate) as a lipid carrier and Pluronic F68 as stabilizer. Surfactant concentration and homogenization speed were taken as optimizing parameters, and the SLN prepared were evaluated by its particle size, zeta potential, entrapment efficiency, surface morphology, and *in vitro* release behavior. The compatibility between drug and lipid was observed using FT-IR, DSC, and XRD studies. Stability studies were conducted for the best formulation for 3 months. Bioavailability studies in male Wistar rats were performed, and pharmacokinetic parameters were assessed using WinNonlin software for SLN and compared with that of pure RL-HCL suspension.

## 2. Materials and Methods

### 2.1. Materials

Raloxifene hydrochloride and Pluronic F68 were obtained as gift samples from Cadila healthcare Ltd., (Gujarat, India); Compritol 888 ATO was obtained as a gift sample from Gattefosse, (SA, France). Dialysis membrane (cutoff MW 12,000–14,000 Daltons) was procured from HiMedia (Mumbai, India). Water used in all experiments was purified by Milli-Q-plus system (Millipore, India). All other chemicals and solvents were of analytical grade.

### 2.2. Preparation of RL-HCL Loaded SLN

RL-HCL loaded SLN were prepared by solvent emulsification/evaporation method. The composition of all the formulations is shown in Table 1. Briefly, 20 mg of drug was dissolved in 10 mL methanol, and 100 mg of lipid was dissolved in 20 mL chloroform separately; drug and lipid solutions were mixed together. The organic solvent mixture was completely evaporated at 70°C using rotary evaporator (IKA RV 10) by purging nitrogen gas to remove the traces of organic solvent [23, 24]. Drug embedded lipid layer was then poured into 100 mL of aqueous solution containing surfactant at 70°C using hot plate and homogenized for 10 minutes at different homogenization speed using high speed homogenizer (IKA UT T25). The suspension was then allowed to cool at room temperature. SLN dispersion was lyophilized using lyophilizer (Decibel Digital Technologies, India) for 36 h at −60°C temperature and pressure below 15 Pascal.

### 2.3. Particle Size, PDI, and Zeta Potential

The particle size was measured by using dynamic light scattering (Delsa Nano C, Beckman coulter), and zeta potential was estimated on the basis of electrophoretic mobility under an electric field [25]. Samples were diluted with distilled water before measurement and measured at a fixed angle of 165° at 25°C for the particle size and polydispersity index (PDI) analysis. For the zeta potential measurement, samples were diluted as 1 : 40 ratio with filtered water (v/v) before analysis. Average particle size, PDI, and zeta potential were then measured in triplicate.

### 2.4. Entrapment Efficiency

Entrapment efficiency (EE) of RL-HCL loaded SLNs was determined by measuring the concentration of unentrapped drug in aqueous medium by centrifugation method [23]. The nanoparticles were centrifuged in a high speed cooling centrifuge (C-24, Remi) using Nanosep centrifuge tubes with ultrafilter having molecular weight cutoff 100 KD (Pall life sciences, India) at 5,000 rpm for 15 min at 4°C, and the supernatant was separated. The amount of RL-HCL in the supernatant was determined at 285 nm using UV-Vis spectrophotometer (U-1800, Hitachi) after appropriate dilution.

The percentage entrapment efficiency (% EE) was calculated by using the following formula:

(1) $$\begin{array}{c} \%\;\text{EE}=\frac{\text{Total}\;\text{drug}\;\text{content}-\text{Free}\;\text{drug}}{\text{Total}\;\text{drug}\;\text{content}}\times 100. \end{array}$$

### 2.5. Surface Morphology

Surface morphology of the best formulation was carried out using scanning electron microscope (SEM) [26]. The formulation was poured into circular aluminum plate and dried in vacuum oven to form a dry film which was then observed under the scanning electron microscope (FEI, Quantum 200E Instrument).

### 2.6. *In Vitro* Release Studies

*In vitro* release studies were performed using pH 6.8 phosphate buffer containing 0.5% v/v polysorbate 80 by dialysis bag method using dialysis membrane having molecular weight of 12,000–14,000 daltons [27]. SLN dispersion equivalent to 0.4 mg of drug was filled into a dialysis membrane bag and tied at both the ends and placed in a beaker containing 100 mL of diffusion medium; temperature and speed were maintained at 37 ± 2°C and 100 rpm, respectively, using magnetic stirrer. Samples were withdrawn at predetermined time intervals, and the same volume was replaced with fresh buffer to maintain the sink condition. Samples were analyzed at 285 nm UV spectrophotometrically. Cumulative percentage release was then calculated from the amount of drug release. The release kinetics were determined by following kinetic equations such as zero order (cumulative % release versus time), first order (log % drug remaining versus time), Higuchi's model (cumulative % drug release versus square root of time), and Korsmeyer-Peppas model (log drug release versus log time). Values of *r* ^2^ and *k* were calculated from the linear curve obtained by regression analysis of the plots. In case of Korsmeyer-Peppas model, *n* value was calculated.

### 2.7. Fourier Transform Infrared Studies

The interaction between drug and lipid was identified from the Fourier transform-infrared (FTIR-8400S, Shimadzu) studies. The FTIR spectrum of RL-HCL, lipid, physical mixture of drug and lipid (1 : 1), and RL-HCL loaded SLN were obtained by the conventional KBr Disc/Pellet method. The samples were prepared by grinding with anhydrous KBr powder and compressed into pellets. The FTIR spectra were measured over the range of 4000–400 cm^−1^ with resolution of 4 cm^−1^ for 50 scans. 

### 2.8. Differential Scanning Calorimetry

Differential scanning calorimetry (DSC) is widely used in thermal analysis to monitor endothermic processes (melting, solid-solid phase transitions, and chemical degradation) as well as exothermic processes (crystallization and oxidative decomposition). It could be extremely useful in preformulation studies since it indicates the existence of possible drug-excipient or excipient-excipient interactions in a formulation. Thermograms of samples (RL-HCL, lipid and physical mixture of drug, and lipid (1 : 1) and RL-HCL loaded SLN) were obtained by using Differential scanning calorimeter (TA Instruments Q 2000). Samples were weighed directly in pierced DSC aluminum pan and scanned in the temperature range of 25–300°C under an atmosphere of dry nitrogen. Heating rate of 10°C/min was used and thermograms obtained were observed for interaction between drug and excipient.

### 2.9. Powder X-Ray Diffractometry

Powder X-ray diffractometric (PXRD) pattern of RL-HCL, lipid, Physical mixture and RL-HCL loaded SLN were obtained by employing X-ray diffractometer (3000, Seifert); Ni-filtered Cu-K radiation, voltage of 40 kV, and current of 30 mA radiation scattered in the crystalline regions were used and measured with a vertical goniometer. Patterns were obtained by using a step size of 0.045°C with a detector resolution in 2*θ* (diffraction angle) between 5° and 80° at 25°C temperature.

### 2.10. Stability Studies

The stability studies of best SLN formulation were performed by being stored at 30° ± 2°C/65% ± 5% relative humidity for 90 days and were examined at regular time intervals for changes in particle size, zeta potential, % EE, and *in vitro* drug release.

### 2.11. Pharmacokinetic Study

#### 2.11.1. Animal Study Protocol

The study protocol was approved by the Central Animal Ethical Committee, Institute of Medical Sciences, Banaras Hindu University, Varanasi, India. Twelve-male Wistar rats of 220 ± 30 grams weight were divided into two groups containing six each (*n* = 6). All rats had free access to water and diet. They were housed in cage; a 12 h dark/light cycle was maintained throughout the study at room temperature (21–24°C), and relative humidity of 50 to 70% was maintained and acclimatized to study area conditions prior to 5 days. General and environmental conditions were strictly monitored. 

#### 2.11.2. Bioanalytical Method

Estimation of RL-HCL in plasma samples was conducted by reversed-phase HPLC [28]. HPLC (Waters, 5.5, USA) setup comprising of binary pump and PDA 2998 detector were used. Mobile phase consisting of acetonitrile, ammonium acetate (pH 4.0, 0.05 M) (50 : 50% v/v) was used at a flow rate of 1 mL/min to elute the drug. The mobile phase was filtered through 0.45 *μ*m nylon filters (Millipore, USA). Samples were injected at 20 *μ*L volume and analyzed at *λ*_max⁡_ 289 nm. 

#### 2.11.3. Oral Administration

The animals were fasted at least 10 h prior to dose administrations and for 4 h after dosing with free access to water. Individual oral doses of the control (RL-HCL suspension) and the test group received the best SLN formulation at a dose of 30 mg/kg [29] body weight. RL-HCL solution equivalent to 30 mg/kg of raloxifene was administered orally into stomach of one group of rats (control group). RL-HCL loaded SLN formulation equivalent to 30 mg/kg of raloxifene was administered orally to stomach in another group of rats (SLN formulation treated). After dosing, rats were anaesthetized with ether and a heparinized capillary was inserted into the retro-orbital vein to get 0.5 mL blood at a time interval of 0.083, 0.25, 0.5, 1.0, 2, 4, 8, 12, and 24 h, respectively. The plasma samples were collected after centrifugation at 5,000 rpm for 15 min and stored immediately at −20°C until analysis. Samples were analyzed by the standardized HPLC method. 

#### 2.11.4. Plasma Sample Preparation

A 100 *μ*L of each plasma sample was transferred into a 1.5 mL polyethylene centrifuge tube. A 50 *μ*L of methanol and 200 *μ*L of acetonitrile were added and vortex-mixed (Remi, Cyclomixer, India) for 1.5 min. The denatured protein precipitation was separated by centrifugation at 15,000 rpm for 10 min at 4°C (Remi Cooling Centrifuge, India). After centrifugation, the supernatant was transferred to fresh tube, and aliquots of 20 *μ*L were directly injected into the HPLC for analysis. 

#### 2.11.5. Pharmacokinetic Data Analysis

Noncompartmental analysis with WinNonlin software Version 4.1, (Pharsight Corp., Mountain View, CA, U.S.A.) was used to estimate the pharmacokinetic parameters (*C*_max⁡_, *T*_max⁡_, AUC, *K*, *t*_1/2_, and MRT) of raloxifene. The maximal plasma concentration of drug (*C*_max⁡_) and the time to reach maximum plasma concentration (*T*_max⁡_) were directly obtained from the observed concentration versus time profiles. 

### 2.12. Statistical Analysis

Results were given as mean ± standard deviation (SD). Mean values of nanoparticles size were compared using the one-way analysis of variance (ANOVA) followed by post Tukey's test. Pharmacokinetic data were analyzed with Student's *t*-test. Differences are considered significant at a level of *P* < 0.05.

## 3. Results and Discussion

### 3.1. Preparation of Raloxifene HCL Loaded SLN

**Figure 1:**
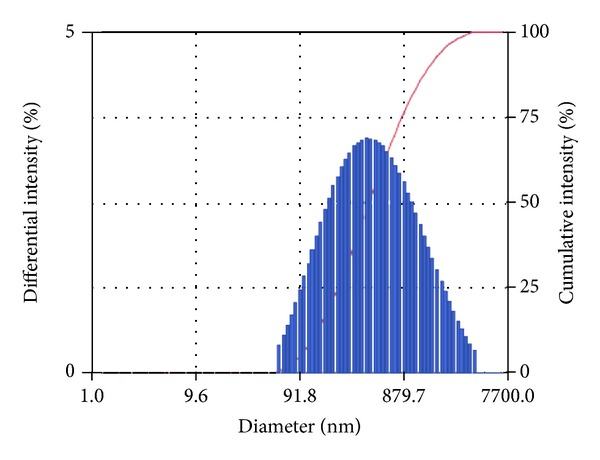
Particle size distribution of best formulation.

Preparation of RL-HCL loaded SLN by solvent emulsification/evaporation method was found to be reliable, simple, and reproducible method. Prepared SLN dispersion was found to be uniform and homogenous in appearance. The particle size of the different batches was found in the range of 274 to 1406 nm. The particle size and PDI of all the formulations are shown in Table 2, and the particle size image of best formulation is shown in Figure 1.

### 3.2. Particle Size and Entrapment Efficiency

#### 3.2.1. Influence of Surfactant Concentration on Particle Size and EE

The effect of surfactant concentrations on particle size and EE was examined by taking three different concentrations of surfactant, that is, 0.5, 1, and 1.5% w/v. It was noted that when the surfactant concentration increases (0.5–1% w/v), the mean particle size was found to decrease sharply. This may be due to insufficient coverage of nanoparticles by the surfactant at lower concentration which results in less stabilization of the particle dispersion [30]. When the surfactant concentration was increased from 1 to 1.5%, there is no significant reduction in the mean particle size. From the results, 1% poloxamer concentration was found to be optimum for stabilizing the SLN dispersion. 

**Figure 2:**
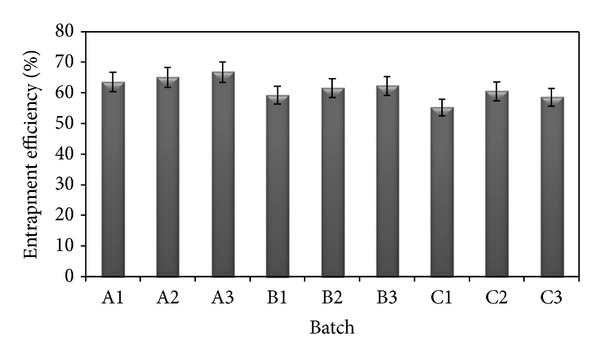
Entrapment efficiency of all formulations.

The EE of different batches is shown in Figure 2. The figure clearly indicates that surfactant concentration significantly affects the EE. In all the three batches of SLN formulations of different homogenization speeds (5,000–15,000 rpm), EE was found to increase with the increase in surfactant concentration (0.5–1.5% w/v) due to increased surface coverage of nanoparticles while increasing the surfactant concentration and thus prevents drug leaching from lipid matrix [30]. 

#### 3.2.2. Influence of Homogenization Speed on Particle Size and EE

The effect of homogenization speed on particle size and EE was observed with three different homogenization speeds, that is, 5,000 rpm, 10,000 rpm, and 15,000 rpm. From the results, it was found that homogenization speed also plays a major role on particle size and EE. The results indicated that when homogenization speed was increased from 5,000 to 15,000 rpm, particle size was found to decrease gradually due to inefficient speed to reduce the particles at lower speed, that is, 5,000 rpm. In case of high speed homogenization, particle size was found to decrease because of the high intensity of shear force acting on the particles which overcomes the intraforces acting in the particles [31]. 

Interestingly, our present work investigated the effect of homogenization speed on entrapment efficiency. At lower homogenization speed, EE was high while at high speed EE was low. The lowering of entrapment efficiency is due to the lack of surfactant molecule with respect to newly generated particles which results in drug diffusion from lipid matrix or small particles have larger surface area to volume ratio causing more drug loss into the SLN dispersion. 

Based on the results of particle size and entrapment efficiency, batch C2 was chosen for further studies as it had lesser particle size (274 nm) than the other batches with higher entrapment efficiency (60.5 ± 1.5%). The zeta potential of best formulation was found to be +16.25. The positive zeta potential might be due to orientation of raloxifene hydrochloride (having one basic nitrogen atom in its structure) on the surface of SLN. 

### 3.3. Morphological Studies

**Figure 3:**
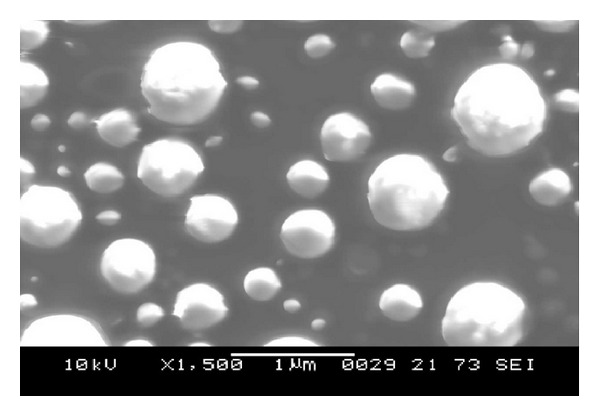
SEM micrograph of best formulation.

The shape and surface morphology of the best formulation and batch C2 were studied by using scanning electron microscopy. The SEM micrograph reveals that the particles were spherical in shape with smooth surface (Figure 3).

### 3.4. *In Vitro* Drug Release Studies

**Figure 4:**
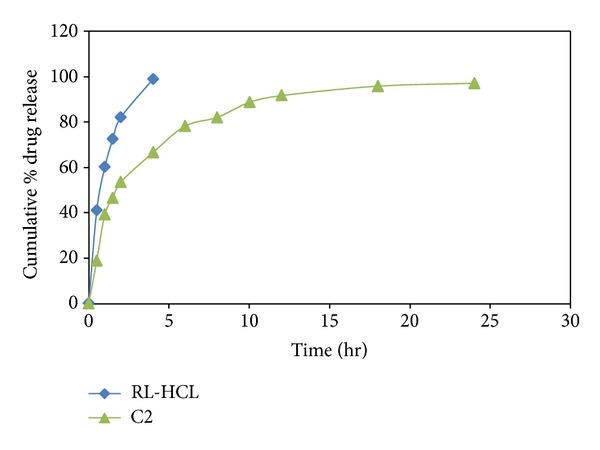
Release profile of best formulation with pure drug.

The *in vitro* release profile of best formulation was compared with pure drug solution (Figure 4). The release of RL-HCL from pure drug solution was higher, nearly 100% release within 4 h, whereas SLN formulation maintained the sustained release of drug up to 24 h. The drug release pattern in SLN formulation showed biphasic release behavior consisting of initial burst release (60% of drug released within 2-3 h) followed by sustained release (nearly 95% of drug was released at 24th h). The initial burst release of the drug is may be due to presence of adsorbed drug on the surface of nanoparticles, and sustained release of drug is may be due to increased diffusional distance and hindering effects by the surrounding solid lipid shell. Release kinetics of best formulation had higher linearity for Higuchi model of release kinetics than the zero-order or first-order kinetics. By the Korsmeyer-Peppas model, “*n*” value was near to 0.5 which indicates that the SLN formulation follows fickian diffusion. 

### 3.5. Fourier Transform Infrared Spectroscopy

**Figure 5:**
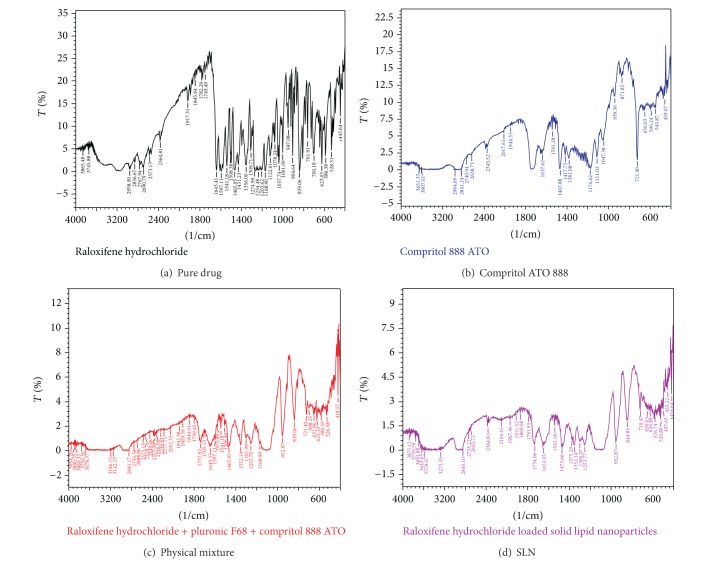
FTIR spectra.

The FTIR spectra of pure drug, lipid, its physical mixture, and SLN formulation were obtained by using FTIR, and the spectra obtained is shown in Figure 5. RL-HCL shows the characteristic peaks at 1643.41 cm^−1^ (C=O stretching), 1597.11 (–C–O–C– stretching), 1465.95 (–S– benzothiophene), and 904.64 (benzene ring). All these peaks were present both in the physical mixture (drug and lipid) and drug loaded SLN, and there is no absence of any functional peaks in all the spectra. Thus, it revealed that there is no significant physicochemical interaction between drug and lipid in the formulation. 

### 3.6. Differential Scanning Calorimetry

**Figure 6:**
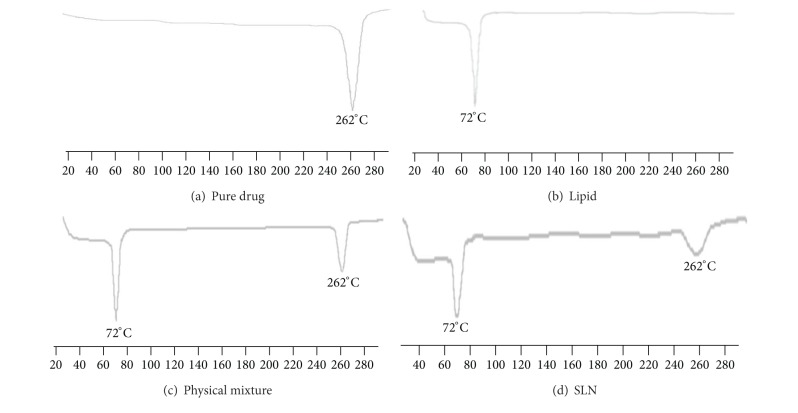
DSC thermograms.

DSC analysis was carried out in order to identify possible interactions between the components. The DSC thermograms of pure drug, lipid, physical mixture, and SLN formulation are shown in Figure 6. RL-HCL and lipid showed a sharp endothermic peak at 262 and 72°C, respectively, due to its crystalline nature. As in the case of physical mixture, thermograms showed the presence of both component sharp peaks, but the thermogram of RL-HCL loaded SLN showed the presence of compritol peak and small broaden drug peak; this could be due to conversion of drug from crystalline to amorphous form in SLN formulation.

### 3.7. PXRD Studies

**Figure 7:**
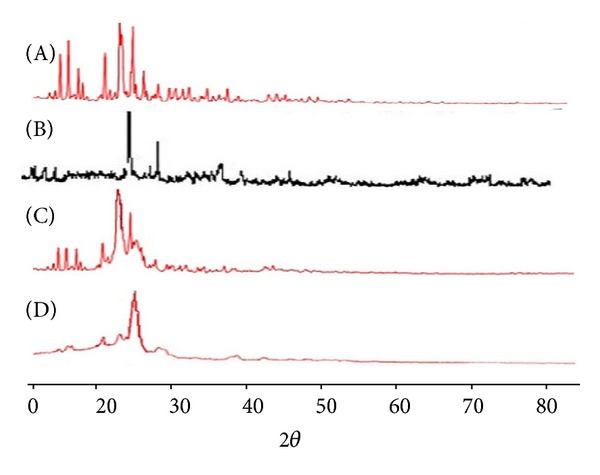
XRD pattern. (A) Pure drug, (B) Lipid, (C) Physical mixture, and (D) SLN.

The PXRD spectra of pure drug, physical mixture, and RL-HCL loaded SLN are shown in Figure 7. The diffraction spectrum of pure RL-HCL showed characteristic peaks at 2*θ* of 13.64, 14.74, 15.97, 19.37, 21.20, 21.57, 22.90, and 24.32, indicating crystalline nature of drug. All the major peaks of RL-HCL were present at the same position in the physical mixture but with reduced intensity. The crystalline peaks of raloxifene were absent in the SLN formulation indicating that the drug was not in crystalline form. Intensity of pure lipid peaks was also decreased in the SLN formulation. This reduced intensity confirms the decreased crystallinity of lipid in SLN formulation. This may be due to the method (solvent evaporation) followed to prepare the SLN formulation.

### 3.8. Stability Studies

The stability study of batch C2 was carried out at 30 ± 2°C/65% ± 5% RH for 90 days. After 90 days, there were no significant change in the particle size, zeta potential, and % EE which reveals that SLN formulation was stable at above conditions (Table 3). The *in vitro* release profile of batch C2 at 0 day and after 90 days showed similar release profile with insignificant difference. There were no differences in the zeta potential after 90 days. From the results, it was confirmed that the SLN formulation had long-term stability; this could be attributed to higher solubility of drug in the lipid matrix and due to poloxamer 188, and because of its nonionic nature it decreases the electrostatic repulsions between the particles, thus stabilizing the nanoparticles by forming a coat around their surfaces.

### 3.9. Pharmacokinetic Data

The HPLC validation parameters of RL-HCL are as follows: the retention time of drug was found to be 9.502 ± 0.61 min, and calibration curve was linear in the concentration range of 0.2–10 *μ*g/mL (*r* ^2^ = 0.995) in phosphate buffer of pH 6.8 and 10–200 ng/mL (*r* ^2^ = 0.995) in rat plasma. The inter- and intraday accuracy and precision was within an R.S.D. ≤2%. LOD, and LOQ were found to be 0.2 and 0.015 *μ*g/mL, respectively. The extraction efficacy in case of spiked plasma samples were 87 ± 17.4, 82 ± 6.9, 91 ± 7.3 (*n* = 3) at concentrations of 0.4, 0.8, and 4.0 *μ*g/mL, respectively.

**Figure 8:**
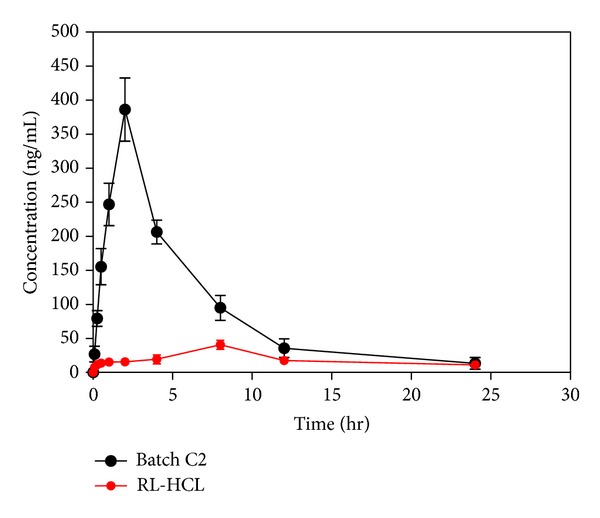
Plasma concentration versus time profile.

The oral bioavailability of RL-HCL is limited due to its poor solubility and extensive first pass metabolism. By considering this, an attempt has been made to improve the bioavailability of RL-HCL by formulating it as SLN. In this study, suspension of RL-HCL and RL-HCL loaded SLN were administered orally to male Wistar rats, and its pharmacokinetic parameters were determined. The plasma concentration versus time profile of RL-HCL suspension and batch C2 is shown in Figure 8, and their mean pharmacokinetic data are also shown in Table 4. The extent of the mean plasma exposures of raloxifene hydrochloride was 5-fold higher in animals treated with RL-HCL loaded SLN compared to animals treated with RL-HCL pure drug. The mean plasma AUC_0–24_ in animals treated with RL-HCL loaded SLN and pure RL-HCL was 2063.26 ± 94.4 ng∗hr/mL and 409.6 ± 34.51 ng∗hr/mL, respectively. This increase in AUC (0–∞) for SLN might be due to the avoidance of first pass metabolism by lymphatic transport. Increase in AUC (0–∞) is may be due to uptake of raloxifene from SLN by Peyer's patch of intestine which is responsible for lymphatic uptake, thus preventing hepatic first pass metabolism [23]. The peak plasma concentration (*C*_max⁡_) value for suspension and SLN formulation were 386.33 ± 80.61 ng/mL and 40.67 ± 6.67 ng/mL. Mean residence times (MRT) were found to be 13.51 ± 1.8 h with pure RL-HCL and 6.68 RL-HCL loaded SLN. This might be due to the controlled release of drug from SLNs. The time to achieve maximum plasma concentration (*T*_max⁡_) was increased from 2.0 hr to 8.0 hr, respectively. These results clearly suggest that the RL-HCL loaded SLN have improved pharmacokinetic profile than the suspension.

## 4. Conclusion

In the present study, RL-HCL loaded SLN were prepared by solvent emulsification/evaporation method. The plasma pharmacokinetics after oral administration of RL-HCL suspension and RL-HCL loaded SLN to male Wistar rats has shown an increased AUC. Bioavailability of RL-HCL loaded SLN was enhanced nearly five times than the pure RL-HCL. Thus, the current experiment results illustrate that SLN as a drug carrier is suitable to increase the bioavailability of RL-HCL.

## 5. Future Perspectives

Various strategies are currently being developed with the aim of enhancing bioavailability of poorly water soluble drugs. Lipid-based drug carriers are one of the promising drug delivery candidates and have wide application for enhancing the bioavailability. Interestingly, our attempt to fabricate SLN of RL-HCL using the simple conventional technique revealed that the use of SLN as a carrier increased the bioavailability of RL-HCL and is found to be fivefold superior to its suspension. Current investigation illustrates that SLN carrier may help for bioavailability enhancement of raloxifene. However, this aspect needs further investigation on exploring the mechanisms behind the lymphatic absorption of SLN. 

## Figures and Tables

**Table 1:** Formulation code for all the batches.

Batch no.	Homogenization speed (1∗1000) rpm	Surfactant concentration (w/v)
A1	5	0.5
A2	5	1.0
A3	5	1.5
B1	10	0.5
B2	10	1.0
B3	10	1.5
C1	15	0.5
C2	15	1.0
C3	15	1.5

Drug (RL-HCL) and lipid (glyceryl behenate) concentration of 20 mg and 100 mg is maintained throughout the study.

**Table 2:** Particle size and PDI of all the formulations.

Batch	Size (nm)	PDI	Zeta potential
A1	1406.3 ± 245.50	1.13 ± 1.05	+24.81
A2	1165.8 ± 58.55	1.26 ± 1.01	+26.12
A3	1101.0 ± 51.05	1.66 ± 2.53	+28.52
B1	925.7 ± 55.80	0.27 ± 0.14	+24.79
B2	631.0 ± 47.75	0.30 ± .028	+20.02
B3	614.3 ± 26.85	0.69 ± 0.88	+20.83
C1	904.1 ± 21.10	0.38 ± 0.12	+29.36
C2	274.3 ± 18.61	0.36 ± 0.08	+16.25
C3	327.4 ± 17.5	0.48 ± 0.14	+18.25

Mean ± SD (*n* = 3).

**Table 3:** Effect of storage time (at 25°C) on particle size, PDI, and entrapment efficiency of SLN (mean ± SD, *n* = 3).

Parameters	Best formulation			
	0 Days	30 Days	60 Days	90 Days
Particle size	271.4 ± 6.35	291.9 ± 3.1	300.3 ± 3.3	329.4 ± 6.65
PDI	0.3 ± 0.02	0.4 ± 0.01	0.473 ± 0.02	0.638 ± 0.02
EE (%)	60.5 ± 1.5	58.3 ± 2.51	57.5 ± 3.05	57.03 ± 3.59

**Table 4:** Pharmacokinetic data of RL-HCL suspension and RL-HCL loaded SLN.

Sample	*T*_max⁡_ (hr)	*C*_max⁡_ (ng/mL)	AUC_0–24_ (ng ∗ hr/mL)	AUC_0–*∞*_ (ng ∗ hr/mL)	MRT_0–*t*_	*t*_1/2_ (hr)
RL-HCL suspension	8.0	40.67 ± 6.67	409.6 ± 34.51	436.66 ± 14.57	13.51 ± 1.8	9.63 ± 2.9
RL-HCL Loaded SLN	2.0	386.33 ± 80.61	2063.26 ± 94.4	2258.3 ± 85.34	6.68 ± 1.1	4.74 ± 1.5

Mean ± SD; *n* = 3.
